# Prospective associations between accelerometry-derived physical activity and sedentary behaviors and mortality among cancer survivors

**DOI:** 10.1093/jncics/pkad007

**Published:** 2023-02-14

**Authors:** Elizabeth A Salerno, Pedro F Saint-Maurice, Fei Wan, Lindsay L Peterson, Yikyung Park, Yin Cao, Ryan P Duncan, Richard P Troiano, Charles E Matthews

**Affiliations:** Division of Public Health Sciences, Department of Surgery, Washington University School of Medicine in St. Louis, MO, USA; Alvin J. Siteman Cancer Center, Washington University School of Medicine in St. Louis, MO, USA; Metabolic Epidemiology Branch, Division of Cancer Epidemiology and Genetics, National Cancer Institute, Rockville, MD, USA; Division of Public Health Sciences, Department of Surgery, Washington University School of Medicine in St. Louis, MO, USA; Alvin J. Siteman Cancer Center, Washington University School of Medicine in St. Louis, MO, USA; Alvin J. Siteman Cancer Center, Washington University School of Medicine in St. Louis, MO, USA; Division of Medical Oncology, Department of Medicine, Washington University School of Medicine in St. Louis, MO, USA; Division of Public Health Sciences, Department of Surgery, Washington University School of Medicine in St. Louis, MO, USA; Alvin J. Siteman Cancer Center, Washington University School of Medicine in St. Louis, MO, USA; Division of Public Health Sciences, Department of Surgery, Washington University School of Medicine in St. Louis, MO, USA; Alvin J. Siteman Cancer Center, Washington University School of Medicine in St. Louis, MO, USA; Division of Gastroenterology, Department of Medicine, Washington University School of Medicine in St. Louis, MO, USA; Program in Physical Therapy, Washington University School of Medicine in St. Louis, MO, USA; Risk Factor Assessment Branch, Epidemiology and Genomics Research Program, Division of Cancer Control and Population Sciences, National Cancer Institute, Rockville, MD, USA; Metabolic Epidemiology Branch, Division of Cancer Epidemiology and Genetics, National Cancer Institute, Rockville, MD, USA

## Abstract

**Background:**

Survival benefits of self-reported recreational physical activity (PA) during cancer survivorship are well-documented in common cancer types, yet there are limited data on the associations between accelerometer-derived PA of all domains, sedentary behavior, and mortality in large, diverse cohorts of cancer survivors.

**Methods:**

Participants included adults who reported a cancer diagnosis in the National Health and Nutrition Examination Survey and wore an accelerometer for up to 7 days in 2003-2006. Participants were followed for subsequent mortality through 2015. We examined the association of light PA, moderate to vigorous PA, total PA, and sedentary behavior, with all-cause mortality. Cox proportional hazards models estimated hazard ratios (HRs) and 95% confidence intervals (CIs), adjusting for demographics and health indicators.

**Results:**

A total of 480 participants (mean age of 68.8 years [SD = 12.4] at the time of National Health and Nutrition Examination Survey assessment) reported a history of cancer. A total of 215 deaths occurred over the follow-up period. For every 1-h/d increase in light PA and moderate to vigorous PA (MVPA), cancer survivors had 49% (HR = 0.51, 95% CI = 0.34 to 0.76) and 37% (HR = 0.63 , 95% CI = 0.40 to 0.99) lower hazards of all-cause mortality, respectively. Total PA demonstrated similar associations with statistically significantly lower hazards of death for each additional hour per day (HR = 0.68, 95% CI = 0.54 to 0.85), as did every metabolic equivalents of task-hour per day increase in total PA estimations of energy expenditure (HR = 0.88, 95% CI = 0.82 to 0.95). Conversely, more sedentary time (1 h/d) was not associated with statistically significantly higher hazards (HR = 1.08, 95% CI = 0.94 to 1.23).

**Conclusions:**

These findings reinforce the current recommendations for cancer survivors to be physically active and underscore the continued need for widespread PA promotion for long-term survival in older cancer survivors.

The number of individuals living beyond a cancer diagnosis, herein referred to as cancer survivors, in the United States is projected to reach 22.2 million by 2030 (1). Consistent and compelling evidence demonstrates the benefits of recreational (ie, leisure-time) physical activity (PA) during long-term survivorship, including improved physical function, cardiorespiratory fitness, and psychosocial health (2-4). Recreational PA is also statistically significantly associated with improved survival after cancer (5); a recent roundtable report from the American College of Sports Medicine reported a consistent inverse association between higher levels of postdiagnosis PA and risk of all-cause mortality (4). However, much of this work is confined to breast, prostate, and colorectal cancer and is based almost entirely on self-reported measures of PA (6).

Self-reported PA assessment is crucial for cost-effective and widespread surveillance and sufficiently estimates MVPA levels at the population level (7). These assessments are prone to bias (8), however, which could contribute to overreporting MVPA and underreporting sedentary behaviors (9) and attenuate their associations with mortality (10). PA questionnaires often focus on MVPA to mirror federal PA guidelines and therefore may not assess light-intensity PA behaviors, such as activities of everyday living (eg, household activities, shopping, caregiving). These lighter-intensity behaviors are often more difficult to recall and report than exercise-specific behaviors. They are also more common during survivorship given renewed cancer-specific recommendations to avoid inactivity if unable to meet PA guidelines (3,11) and high rates of cancer-related fatigue (12) that may limit participation in more strenuous activities. Understanding the potential survival benefits associated with multiple PA intensities (ie, light, MVPA, total) is an important and necessary step toward personalized PA recommendations after cancer.

One solution to the limitations of self-reported PA assessments is to use accelerometers. Waist-worn monitors capture bodily acceleration that is summarized over specific epochs (eg, 1 minute), which can then characterize the intensity, duration, and total volume of daily activity. The National Health and Nutrition Examination Survey (NHANES) 2003-2004 and 2005-2006 cycles included accelerometry in representative samples of US adults and followed individuals for subsequent mortality. Thraen-Borowski and colleagues (13) recently reported that cancer survivors engaged in less accelerometry-derived light PA and MVPA and more sedentary behavior than matched adults, which is consistent with previous NHANES analyses in cancer survivors (14-18).

To date, there is no comprehensive analysis, to our knowledge, of the relationship between accelerometry-derived PA and sedentary behavior and mortality among cancer survivors in a representative sample of US adults. To address this gap, we investigated if PA of other intensities (ie, not just MVPA) and sedentary behavior were associated with mortality in NHANES cancer survivors. With over 10 years of follow-up, multiple indicators of health status, and a wide variety of cancer types, this analysis represents a prime opportunity to advance our understanding of the relationships between PA, sedentary behaviors, and all-cause mortality among long-term cancer survivors.

## Methods

### Participants and study design

NHANES collects extensive health data from a representative sample of US adults (19). Details on data collection in NHANES have been reported elsewhere (20,21). Briefly, NHANES 2003-2004 and 2005-2006 included a representative sample of noninstitutionalized US adults. Between 2003 and 2006, participants were assessed for PA levels and self-reported up to 4 cancer diagnoses and corresponding ages at diagnosis. The first chronological cancer reported was considered the primary cancer (n = 630). Nonmelanoma skin primary cancers were removed (n = 150), leaving a final analytic sample of 480 adult cancer survivors (Table 1). Participants were followed for subsequent mortality through December 2015. NHANES protocols were approved by the National Center for Health Statistics ethics review board, and all participants provided written informed consent. This analysis was not subject to institutional review board review based on National Institutes of Health policy because it consisted of deidentified data with no direct participant contact; thus, it is not human subjects research.

**Table 1.** Distribution of cancer types in current sample of US cancer survivors in NHANES 2003-2006

Cancer type	Frequency No. (%)
Breast	102 (21.3)
Prostate	93 (19.4)
Other^a^	63 (13.1)
Colon	40 (8.3)
Melanoma	40 (8.3)
Cervical	37 (7.7)
Uterine	25 (5.2)
Lymphoma	14 (2.9)
Multiple primaries^b^	14 (2.9)
Lung	13 (2.7)
Bladder	11 (2.3)
Kidney	11 (2.3)
Thyroid	11 (2.3)
Unknown/missing	6 (1.3)

a Cancer types with fewer than 10 participants were collapsed into 1 category. NHANES = National Health and Nutrition Examination Survey.

b Participants reporting multiple cancers diagnosed at the same time were collapsed into 1 category.

### Measures

#### Accelerometry

From 2003 to 2006, participants were asked to wear an Actigraph model 7164 accelerometer on the nondominant hip during all waking hours for a 7-day period (19). PA data retained for analysis met wear time validation criteria of at least 10 hours of wear time per day for at least 1 day, with nonwear time defined using an automated algorithm (19). Sedentary time was defined as hours per day spent below 100 counts per minute (cpm), and total PA time was defined as hours per day spent at or above 100 cpm (22). Light-intensity PA was defined as hours per day between 100 and 759 cpm, and MVPA was defined as hours per day spent at or greater than 760 cpm (23). We further explored estimations of energy expenditure, calculated from metabolic equivalents of task (MET) hours using the Freedson equation [METS/min = 1.439008 + 0.000795 × count/min (vertical axis)] (24) and total PA as time recorded above 100 cpm.

#### Mortality

All-cause mortality was assessed through linkage to the National Death Index through December 31, 2015 (25). International Classifications of Diseases (ICD)-9 and ICD-10 codes were used to classify deaths due to all causes. Person-years accrued from the interview date to the date of death or censoring (December 31, 2015), whichever came first.

#### Covariates

Covariates included age, sex, race, education, smoking and alcohol status, body mass index (BMI), diet quality, chronic conditions, mobility, health status, frailty, cancer type, and time since diagnosis. Demographic information (age, sex, education), health behaviors (smoking status), and diagnoses of chronic conditions (diabetes, heart disease, heart failure, stroke, chronic bronchitis, emphysema) were self-reported. Race and ethnicity were also self-reported using fixed categories (Mexican American, Non-Hispanic Black, Non-Hispanic White, Other Hispanic, and Other [including Alaska Native, Asian, other Hispanic, and other race and ethnicity including multiracial]) to characterize the population and oversample Mexican American and Non-Hispanic Black adults. Height and weight were measured, and BMI was calculated using the standard kg/m^2^ equation. Diet quality was measured using 24-hour recall measures of 12 dietary components from the Healthy Eating Index-2005 (26) (range 0-100; higher scores indicate healthier diet). Mobility limitations were assessed through reported difficulty walking 0.25 miles without special equipment or up 10 steps in adults aged at least 60 years. Participants younger than 60 years were assessed for mobility limitations if they reported limitations related to work, memory problems, or other physical or mental limitations. Self-reported health was measured with the question “Would you say your health in general is excellent, very good, good, fair, or poor?” A frailty index was created based on the concept of deficit accumulation (27) and has been described in detail elsewhere (28). Briefly, this index was derived from 38 self-reported and clinically assessed health indicators. Items were summed and divided by the number of available items; individuals were then categorized as robust (≤0.10), vulnerable (0.11 to 0.21), frail (0.22 to 0.45), or most frail (>0.45) (29). Time since diagnosis was calculated by subtracting current age from the age reported at primary cancer diagnosis.

### Statistical analysis

Cox proportional hazards regression models were used to estimate hazard ratios (HRs) and 95% confidence intervals (CIs), adjusting for covariates based on previous research (28,30), and included age, sex, race and ethnicity, education, diet, smoking status, BMI, self-reported health, mobility limitations, and diagnoses of diabetes, stroke, heart disease, heart failure, chronic bronchitis, and emphysema. Missingness for any given covariate was minimal (≤5%) and thus treated as missing in all models. The proportional hazards assumption for key exposures was graphically checked using Schoenfeld residual and Kaplan-Meier plots. PA variables were modeled both continuously via 1-hour intervals per day and categorically via quartiles.

To examine possible effect modification, we conducted stratified analyses by sex (male, female), age at time of NHANES assessment (median split at 71 years), BMI (<25, 25 to <30, ≥30), frailty status (robust or vulnerable, frail or most frail), health status (very good or excellent, good, fair or poor), mobility limitations (yes, no), chronic conditions (0, ≥1), weight change over the past year (losers, gainers or maintainers), time from diagnosis to accelerometry monitoring (≤2 years, >2 years), and time from monitoring to death (<5 years, ≥5 years). Further, post hoc sensitivity analyses investigated the possibility of reverse causality, as recommended by Strain and colleagues (31), through exclusions of individuals who 1) reported 1 or more chronic condition; 2) reported 2 or more chronic conditions; 3) were most frail; 4) lost weight over the past year; 5) died within 1 year of monitoring, and; 6) died within 2 years of monitoring. Finally, we explored differential associations by cancer type (major: breast, prostate, colon; minor: all others) using both exclusions and stratifications (32). Total PA was used for post hoc sensitivity analyses, measured continuously. All analyses were conducted in SAS 9.4 and SUDAAN, incorporating sample weights as recommended by the National Center for Health Statistics (33) to account for survey cycles, strata, and sampling units; statistical significance was set at .05.

## Results

Table 2 details participant characteristics. On average, participants were overweight (BMI mean = 27.9 kg/m^2^, SD = 5.8) and frail (63.9% frail or most frail status) with a common cancer (49.0% breast, prostate, or colon) (Table 2). Participants were followed-up for an average of 12.0 (SD = 11.8) years after their cancer diagnosis, and average follow-up time between monitoring and death or censoring was 8.4 (SD = 3.7) years (Table 2). A total 215 deaths occurred between monitoring and death or censoring. Compared with those in the lowest quartile of MVPA, participants in the highest quartile were statistically significantly younger (*P* < .001), less frail (*P* < .001), and healthier (eg, fewer mobility limitations and chronic conditions). Participants in the highest quartile of sedentary behavior were more likely to be older (>71 years; *P* < .001), male (*P* = .001), frailer (*P* < .001), and less healthy (eg, more mobility limitations and chronic conditions) compared with those in the lowest quartile.

**Table 2.** Descriptive demographic and cancer-specific characteristics of US cancer survivors in NHANES 2003-2006^a^

Characteristic		Light PA			MVPA			Sedentary behavior		
	Full sample, %	Lowest quartile, % (n = 120)	Highest quartile, % (n = 120)	*P*	Lowest quartile, % (n = 121)	Highest quartile, % (n = 120)	*P*	Lowest quartile, % (n = 121)	Highest quartile, % (n = 120)	*P*
	(n = 480)									
Mean age (SD), y	68.8 (12.4)	74.9 (9.0)	65.9 (12.8)	<.001	75.9 (8.9)	60.7 (11.7)	<.001	62.2 (12.8)	73.3 (10.3)	<.001
Mean BMI^a^ (SD), kg/m^2^	27.9 (5.8)	28.1 (6.5)	27.7 (5.8)	.58	27.5 (6.2)	27.5 (5.1)	.46	27.9 (5.5)	27.9 (6.6)	.36
Race and ethnicity				.86			.05			.002
Mexican American	5.4	4.2	7.5		3.3	6.7		10.7	1.7	
Non-Hispanic										
Black	15.6	14.2	16.7		18.2	14.2		13.2	21.7	
White	74.8	79.2	70.8		72.7	75.8		71.9	72.5	
Other Hispanic	1.3	0.8	1.7		0.8	1.7		1.7	0.8	
Other groups^b^	2.9	1.7	3.3		5.0	1.7		2.5	3.3	
Sex				.05			.95			.001
Female	55.0	49.2	61.7		56.2	55.8		66.9	46.7	
Male	45.0	50.8	38.3		43.8	44.2		33.1	53.3	
Self-reported health status				.36			.08			.47
Excellent	6.9	6.7	3.3		6.6	9.2		5.8	9.2	
Very good	24.4	19.2	26.7		15.7	28.3		22.3	20.8	
Good	37.5	31.7	44.2		30.6	36.7		37.2	35.8	
Fair/poor	26.7	38.3	21.7		41.3	20		29.8	30.8	
Frailty index				<.001			<.001			<.001
Robust	4.8	1.7	3.3		1.7	12.5		9.9	1.7	
Vulnerable	31.3	19.2	40.8		11.6	50.0		38.8	24.2	
Frail	51.0	53.3	45.8		57.9	34.2		44.6	55.8	
Most frail	12.9	25.8	10.0		28.9	3.3		6.6	18.3	
History of										
Congestive heart disease	9.4	17.5	5.8	<.01	18.2	5.8	.01	7.4	14.2	.09
Stroke	8.5	14.2	8.3	.15	12.4	6.7	.13	4.1	8.3	.18
Diabetes	15.2	24.2	15.8	.11	24.0	8.3	.001	11.6	20.0	.05
Heart failure	8.3	13.3	6.7	.59	15.7	4.2	.59	5.8	12.5	.90
Chronic bronchitis	12.5	11.7	14.2	.57	15.7	10.8	.31	9.9	15.0	.23
Emphysema	7.5	12.5	4.2	.04	14.0	5.0	.27	4.1	10.0	.08
Mobility limitations	38.8	57.5	29.2	<.001	65.3	16.7	<.001	28.9	51.7	.01
Chronic conditions				<.001			<.001			.03
0	66.9	54.2	67.5		55.4	76.7		72.7	59.2	
≥1	33.1	45.8	32.5		44.6	23.3		27.3	40.8	
Weight change				.19			.09			.09
Losers	37.4	39.2	31.7		43.0	32.5		34.7	44.2	
Gainers/maintainers	62.6	58.3	67.5		57.0	67.5		65.3	55.8	
Mean time between diagnosis and monitoring (SD), y	12.0 (11.8)	12.7 (12.7)	12.8 (11.2)	.97	12.7 (12.0)	12.1 (10.3)	.65	11.8 (11.0)	12.9 (13.1)	.49
Mean time between monitoring and death (SD), mo	8.4 (3.7)	6.7 (3.9)	9.3 (3.1)	<.001	6.2 (3.9)	10.0 (2.6)	<.001	9.6 (3.0)	7.7 (3.6)	<.001

a BMI = body mass index; MVPA = moderate to vigorous physical activity; NHANES = National Health and Nutrition Examination Survey; PA = physical activity; SD = standard deviation.

b Other groups include Alaska Native, Asian, other Hispanic, or other race and ethnicity including multiracial.

### PA and sedentary behavior

**Figure 1.**
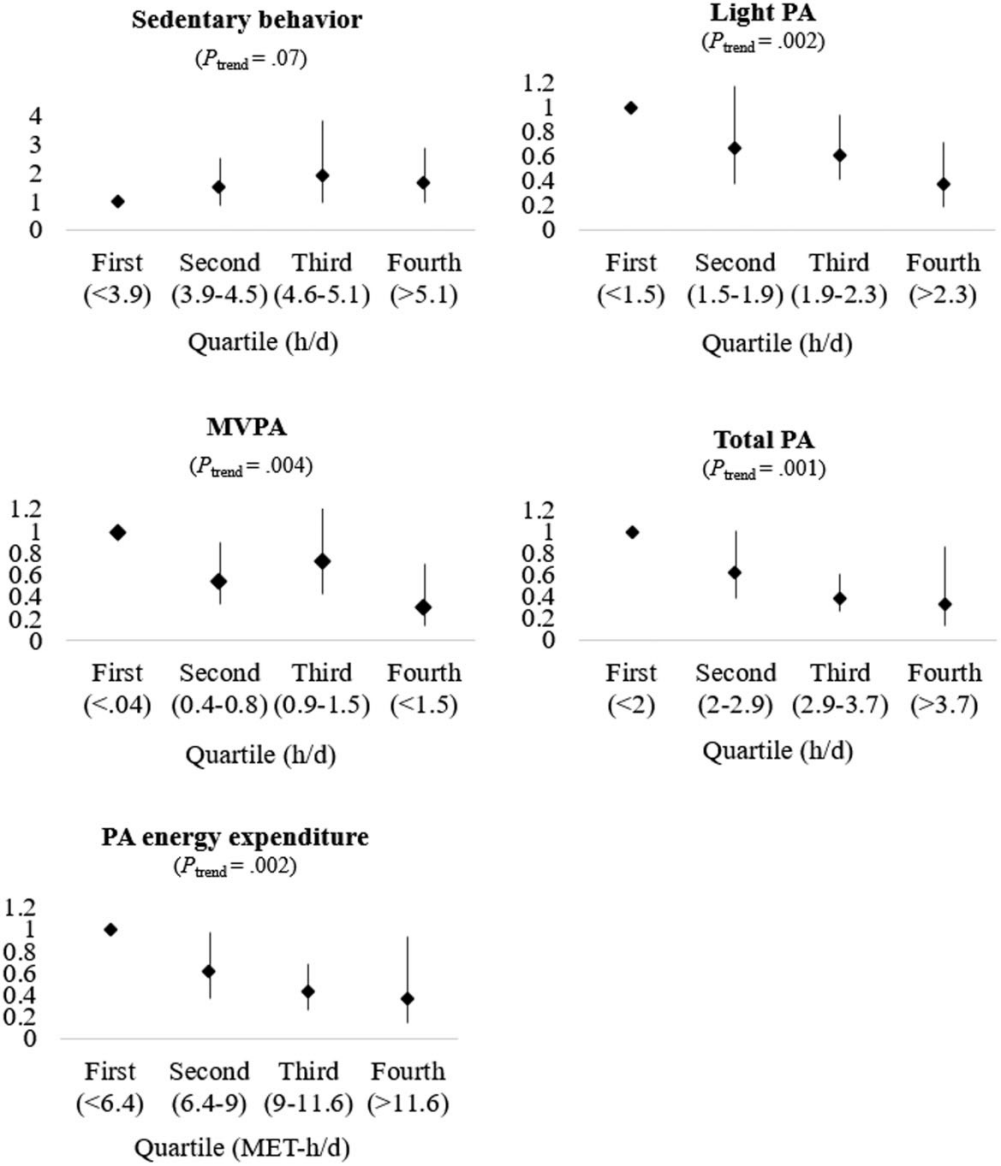
Mortality hazard ratios by quartiles of sedentary behavior and physical activity among US cancer survivors in National Health and Nutrition Examination Survey (NHANES) 2003-2006. All multivariable models adjusted for age, sex, race and ethnicity, education, diet, smoking status, body mass index, self-reported health, mobility limitations, frailty, time since diagnosis, primary cancer type, and diagnoses of diabetes, stroke, heart disease, heart failure, chronic bronchitis, and emphysema. MVPA = moderate to vigorous physical activity; PA = physical activity.

Table 3 details hazard ratios and 95% confidence intervals for age-adjusted, multivariable, and MVPA-adjusted models. Figure 1 depicts the quartile associations with *P* trends. More light-intensity PA was associated with statistically significantly lower risk of death, such that mortality risk was lower with each increasing hour (HR = 0.51, 95% CI = 0.34 to 0.76) and quartile (*P*_trend_ = .002) of light PA. MVPA demonstrated similar associations, both continuously for every additional hour of MVPA (HR = 0.63, 95% CI = 0.40 to 0.99) and categorically via quartiles (*P*_trend_ = .004). When exploring these associations by total PA, each increasing hour per day of activity of any intensity was associated with statistically significantly lower hazards of death (continuous HR = 0.68, 95% CI = 0.54 to 0.85, *P*_quartile trend_ = .001). These findings persisted for every additional MET-hour per day estimates of PA energy expenditure (continuous HR = 0.88, 95% CI = 0.82 to 0.95, *P*_quartile trend_ = .002). Conversely, each 1-h/d increase in sedentary behavior was not associated with statistically significantly higher mortality hazards (HR = 1.08, 95% CI = 0.94 to 1.23, *P*_quartile trend_ = .07). Further mutual adjustment for continuous daily hours of MVPA did not substantially attenuate mortality associations for sedentary behavior, light PA, or PA energy expenditure (Table 3). Finally, a model with sedentary behavior, light PA, and MVPA indicated a statistically significant independent effect of light PA on mortality (HR = 0.55, 95% CI = 0.35 to 0.87).

**Table 3.** Associations between sedentary behavior or physical activity and mortality among US cancer survivors in NHANES 2003-2006

Accelerometer variable	Age-adjusted^a^	Multivariable^b^	MVPA adjustment^c^	Deaths, No.	Cases, No.
	HR (95% CI)	HR (95% CI)	HR (95% CI)		
Sedentary behavior (h/d)					
Continuous	1.26 (1.11 to 1.42)	1.08 (0.94 to 1.23)	1.01 (0.88 to 1.16)	215	480
First quartile (<3.9)	1.0	1.0	1.0	30	121
Second quartile (3.9-4.5)	1.60 (0.84 to 3.04)	1.49 (0.88 to 2.53)	1.31 (0.80 to 2.15)	49	119
Third quartile (4.6-5.1)	1.90 (1.00 to 3.61)	1.90 (0.94 to 3.85)	1.58 (0.80 to 3.12)	63	120
Fourth quartile (>5.1)	2.22 (1.18 to 4.19)	1.68 (0.97 to 2.89)	1.33 (0.81 to 2.18)	73	120
Light PA (h/d)					
Continuous	0.50 (0.37 to 0.67)	0.51 (0.34 to 0.76)	0.55 (0.35 to 0.88)	215	480
First quartile (<1.5)	1.0	1.0	1.0	86	120
Second quartile (1.5-1.9)^d^	0.59 (0.33 to 1.04)	0.66 (0.37 to 1.18)	0.70 (0.38 to 1.29)	51	120
Third quartile (1.9-2.3)^d^	0.51 (0.34 to 0.76)	0.62 (0.41 to 0.94)	0.70 (0.43 to 1.16)	41	120
Fourth quartile (>2.3)	0.38 (0.24 to 0.61)	0.37 (0.19 to 0.72)	0.43 (0.21 to 0.87)	37	120
MVPA (h/d)					
Continuous	0.52 (0.35 to 0.77)	0.63 (0.40 to 0.99)	—	215	480
First quartile (<0.4)	1.0	1.0	—	89	121
Second quartile (0.4-0.8)	0.53 (0.37 to 0.76)	0.54 (0.33 to 0.90)	—	59	119
Third quartile (0.9-1.5)	0.52 (0.29 to 0.90)	0.72 (0.42 to 1.21)	—	45	120
Fourth quartile (>1.5)	0.24 (0.12 to 0.46)	0.31 (0.14 to 0.70)	—	22	120
Total PA (h/d)			—		
Continuous	0.62 (0.56 to 0.69)	0.68 (0.54 to 0.85)	—	215	480
First quartile (<2)	1.0	1.0	—	90	120
Second quartile (2-2.9)^d^	0.62 (0.43 to 0.90)	0.62 (0.38 to 1.01)	—	62	121
Third quartile (2.9-3.7)^d^	0.35 (0.22 to 0.57)	0.39 (0.25 to 0.61)	—	38	119
Fourth quartile (>3.7)	0.29 (0.17 to 0.52)	0.33 (0.13 to 0.86)	—	25	120
PAEE (MET-h/d)					
Continuous	0.86 (0.81 to 1.08)	0.88 (0.82 to 0.95)	0.83 (0.72 to 0.96)	215	480
First quartile (<6.4)	1.0	1.0	1.0	91	120
Second quartile (6.4-9)^d^	0.58 (0.38 to 0.88)	0.61 (0.38 to 0.98)	0.61 (0.37 to 1.01)	59	120
Third quartile (9-11.6)^d^	0.37 (0.23 to 0.57)	0.43 (0.27 to 0.69)	0.45 (0.24 to 0.84)	39	120
Fourth quartile (>11.6)	0.29 (0.17 to 0.47)	0.37 (0.15 to 0.93)	0.40 (0.08 to 1.88)	26	120

a Age-adjusted models adjusted for age only. CI = confidence interval; HR = hazards ratio; MVPA = moderate to vigorous physical activity; NHANES = National Health and Nutrition Examination Survey; PA = physical activity; PAEE = physical activity energy expenditure.

b Multivariable models adjusted for age, sex, race and ethnicity, education, diet, smoking status, body mass index, self-reported health, mobility limitations, frailty, time since diagnosis, primary cancer type, and diagnoses of diabetes, stroke, heart disease, heart failure, chronic bronchitis, and emphysema.

c MVPA adjustment models adjusted for all multivariable variables plus continuous moderate to vigorous physical activity. Results are not presented for MVPA and Total PA, as MVPA is already included in those exposures.

d Numbers are rounded.

### Sensitivity analyses

**Figure 2.**
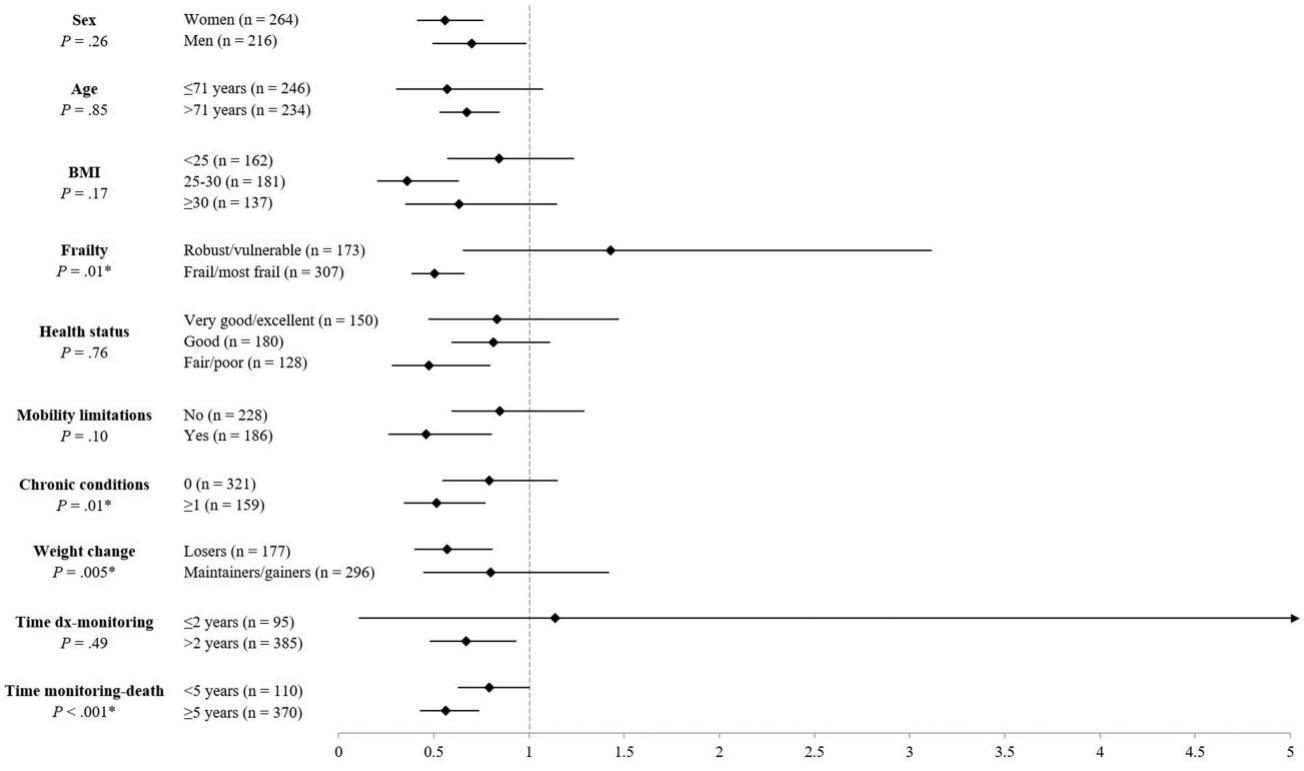
Stratified associations between total physical activity and mortality among US cancer survivors in the National Health and Nutrition Examination Survey (NHANES) 2003-2006. All multivariable models adjusted for age, sex, race and ethnicity, education, diet, smoking status, body mass index, self-reported health, mobility limitations, frailty, time since diagnosis, primary cancer type, and diagnoses of diabetes, stroke, heart disease, heart failure, chronic bronchitis, and emphysema. BMI = body mass index; Dx = diagnosis.

Stratified analyses indicated statistically significantly different associations between total PA and mortality by several indicators. Specifically, cancer survivors who were frail or most frail, had 1 and more chronic conditions, lost weight in the past year, and were at least 5 years from monitoring demonstrated greater mortality protection with increasing levels of PA (*P*_interactions_ < .01). No statistically significant interactions emerged for sex, age, BMI, health status, mobility limitations, or time between diagnosis and monitoring (*P*_interactions_ > .10) (Figure 2). Further post hoc sensitivity analyses investigated the possibility of reverse causation through exclusionary analyses. Table 4 details hazard ratios and 95% confidence intervals in the original full sample and after varying exclusions (eg, chronic conditions, died within 1 year). These analyses suggest that our findings of reduced mortality risk with increased levels of PA are robust, if slightly inflated (Table 4). Finally, sensitivity analyses in major cancer types only (eg, breast, prostate, colon; n = 235, deaths = 116) confirmed the association between total PA and all-cause mortality (HR = 0.74, 95% CI = 0.58 to 0.94). Further stratification confirmed that this association did not differ by major vs minor cancer type (*P *=* *.35).

**Table 4.** Post hoc sensitivity analyses to explore reverse causation using total physical activity among US cancer survivors in NHANES 2003-2006^a^

Sensitivity analysis	No. of cases removed	No. of deaths	HR (95% CI)
Original sample (n = 480)	0	215	0.68 (0.54 to 0.87)
Exclusions			
≥1 chronic conditions	159	121	0.82 (0.55 to 1.23)
≥2 chronic conditions	53	177	0.72 (0.55 to 0.94)
Most frail	65	167	0.71 (0.58 to 0.88)
Lost weight	177	128	0.63 (0.46 to 0.86)
Died within 1 year	23	192	0.73 (0.59 to 0.90)
Died within 2 years	44	171	0.66 (0.52 to 0.84)

a All models adjusted for age, sex, race and ethnicity, education, diet, smoking status, body mass index, self-reported health, mobility limitations, frailty, time since diagnosis, primary cancer type, and diagnoses of diabetes, stroke, heart disease, heart failure, chronic bronchitis, and emphysema. CI = confidence interval; HR = hazard ratio; NHANES = National Health and Nutrition Examination Survey.

## Discussion

To our knowledge, this is the first analysis exploring the associations between accelerometry-derived PA, sedentary behavior, and all-cause mortality in a national sample of cancer survivors. Light PA, MVPA, and total PA were statistically significantly associated with a lower risk of mortality. Higher levels of sedentary behavior were not statistically significantly associated with greater mortality risk. Overall, these findings confirm the benefits of varying PA intensities after a cancer diagnosis, particularly in older adult cancer survivors, and underscore the importance of continued PA promotion during long-term survivorship.

Our results are consistent with the literature documenting lower mortality risk with higher levels of self-reported PA during cancer survivorship, both generally (4,5,32) and in NHANES specifically (6). The current analysis extends this work to include accelerometry-derived measures and explores a wider range of PA intensities than are traditionally captured in self-report. Our findings highlight the importance of lighter-intensity activities, and total PA accumulation, for survival benefits, even after mutual adjustment for MVPA. More recent work has highlighted the positive relationship between light PA and survival in the general population (34-36), suggesting that a broader range of intensities may promote longevity. We further demonstrated that this association may be independent of sedentary behavior and MVPA in this sample of cancer survivors. Such findings are important in this older adult cancer population due to the unique confluence of barriers (eg, fatigue, neuropathy) to initiating and maintaining PA regimens (37). Although separating out actionable domain- and intensity-specific PA will be important to guide survivors in meeting cancer-specific guidelines (3), it is encouraging to see survival associations with total accumulated PA, not just recreational. Future PA programs may focus on a wider range of intensities to tailor exercise prescriptions while maintaining survival benefits in this diverse population.

Sedentary behavior has distinct facilitators and barriers from PA (38) and has emerged as a strong predictor of premature mortality (39-41). In cancer survivors specifically, increased sedentary behavior has been associated with poor quality of life, pain, and fatigue (42,43). The epidemiological association between sedentary behavior and cancer mortality is more limited, with only a few studies reporting a modest 12%-13% higher risk of cancer mortality in most vs least time spent sedentary (4). However, these studies have included both adults with and without cancer and used self-reported measures of sedentary behavior. Our current findings did not demonstrate statistically significantly higher mortality risk with more sedentary time, which may be due to suboptimal power. Though not statistically significant, the elevated hazard ratio is consistent with self-reported sedentary behavior-mortality findings in NHANES cancer survivors (6). Given its hip placement, it is possible that the accelerometer misclassified certain stationary but light-intensity activities (eg, standing) as sedentary. Other devices, such as the thigh-worn activPAL (PAL Technologies Ltd), may be better suited to measure sedentary behavior (44). Regardless, the sedentary behavior hazard ratio is consistent with other studies (4,6) and warrants future confirmatory studies. These studies should also consider if and how sedentary behavior offsets PA during survivorship, as seen in the general population (45), and how the behavioral tradeoff of reducing sedentary behavior to increase light PA, and vice versa (46,47), may be associated with mortality.

Given stronger associations for the protective role of PA in individuals with poorer health, overestimation of the strength of associations is a concern. Indeed, accelerometry-mortality analyses in the general population may be subject to reverse causation (28), particularly in samples with short follow-up (<6 years), older participants (≥65 years of age), and limited statistical adjustment for poor health at the time of accelerometry measurement. The current NHANES analysis includes over 10 years of follow-up and multiple indicators of health status, providing a unique opportunity to explore these relationships while minimizing potential confounding. We conducted several stratified and post hoc analyses to test for reverse causation and largely confirmed our findings with consistent strength of associations (Table 4). However, we have previously discussed (28) that the reverse causation phenomenon in the context of accelerometry-derived PA, also known as “confounding by health status,” is not directly quantifiable and can only be inferred through comparisons of hazards ratios across models where confounding may be more or less present. Given that our sample included a subgroup of cancer survivors in NHANES with further reduced sample sizes in stratified analyses, we cannot completely rule out the possibility of this bias. Replicating these findings in larger cancer cohorts of varying ages with additional confounding variables will be important to confirm the PA-mortality association.

There are several strengths and limitations of this analysis. We leveraged a cohort of a representative sample of noninstitutionalized adults in the United States with prospective linkage to death indices, which allowed us to characterize the association between accelerometry-derived PA and sedentary behaviors with mortality in 480 adult cancer survivors. This analysis extends work that has previously been confined to self-reported PA measures (32,48) and common cancer sites (49,50). Our sensitivity analyses suggest that the PA survival benefits are not limited to breast, prostate, and colorectal cancers (4); however, these cancer types still represented most cases in the current sample. Due to small sample sizes after stratification, we were unable to robustly explore these associations within cancer types. This will be important to determine if these associations are consistent across disease sites, as suggested herein, or rather differential. Although 7 days of monitoring is reflective of usual PA levels of months and years (51,52), and the strength of the PA accelerometer-mortality association appears to hold over many years in the general NHANES population (28), it is possible that the lack of updated PA information during follow-up could reduce the strength of associations observed. Prediagnosis PA was also not available for analysis as a covariate. Cancer survivors in this sample were more than a decade postdiagnosis on average and thus had survived long enough to be part of such an analysis. There was a large presence of older age (eg, ≥65 years) and frailty in our sample, suggesting that these findings are most salient for older adult cancer survivors. Investigators should attempt to reproduce these findings in individuals closer to diagnosis and/or treatment to better understand these relationships in the acute stages of disease. We also had limited information on cancer stage, treatment regimen, or other cancer-specific clinical characteristics; it is possible that individuals who receive more intensive treatment have different behavioral and clinical profiles and thus different survival patterns.

In conclusion, our results support the beneficial association between PA and survival in long-term, older adult cancer survivors. Importantly, a broad range of PA intensities was associated with reduced risk of all-cause mortality. Promoting PA and discouraging sedentary behaviors after a cancer diagnosis should be a priority for providers and researchers alike, with renewed focus on lighter-intensity activities for those individuals who may not be able or willing to engage in higher-intensity exercise.

## Data Availability

The data that support the findings of this study are available on the Centers for Disease Control and Prevention’s webpage at https://www.cdc.gov/nchs/nhanes/index.htm. These data were derived from the following resources available in the public domain: https://wwwn.cdc.gov/nchs/nhanes/continuousnhanes/default.aspx?BeginYear=2003; https://wwwn.cdc.gov/nchs/nhanes/continuousnhanes/default.aspx?BeginYear=2005.
